# Solitary and multiple thyroid nodules as predictors of malignancy: a systematic review and meta-analysis

**DOI:** 10.1186/s13044-022-00140-6

**Published:** 2022-12-05

**Authors:** Aqeeb Ur Rehman, Muhammad Ehsan, Haseeba Javed, Muhammad Zain Ameer, Aleenah Mohsin, Muhammad Aemaz Ur Rehman, Ahmad Nawaz, Zunaira Amjad, Fatima Ameer

**Affiliations:** 1 Department of Medicine, King Edward Medical University, Lahore, Pakistan; 2 Clinical Research Fellow, Massachusetts General Hospital, Harvard Medical School, Boston, USA; 3 Department of Medicine, Services Institute of Medical Sciences, Lahore, Pakistan; 4 Department of Medicine, Mayo Hospital, Lahore, Pakistan

**Keywords:** Thyroid nodule, Thyroid cancer, Thyroid carcinoma, Single thyroid nodule, Single nodule, Multinodular goiter, Nodular goiter

## Abstract

**Background:**

The debate on whether or not there is a difference in the incidence of thyroid cancer between the patients with Solitary thyroid Nodule (STN) and Multinodular Goiter (MNG) has been constantly present for the last few decades. With newer studies yielding mixed results, it was imperative to systematically compile all available literature on the topic.

**Methods:**

PubMed/MEDLINE, Cochrane Central, ScienceDirect, GoogleScholar, International Clinical Trials registry, and reference lists of the included articles were systematically searched for article retrieval. No filter was applied in terms of time, study design, language or country of publication. Rigorous screening as per PRISMA guidelines was undertaken by 2 independent reviewers in order to identify the articles that were most relevant to the topic.

**Results:**

Twenty-two studies spanning from 1992 to 2018 were included in this analysis and encompassed 50,321 patients, 44.2% of which belonged to the STN subgroup and 55.37% to the MNG subgroup. MNG was found to be associated with a significantly lower risk of thyroid cancer (OR = 0.76; 95% CI 0.61–0.96) when compared with STN. Papillary carcinoma was the most frequently occurring carcinoma across both groups, followed by follicular and medullary carcinomas. A subgroup analysis was performed to assess the efficacy of the two most commonly employed diagnostic tools i.e. surgery and fine needle aspiration cytology (FNAC), however it yielded nonsignificant results, indicating a comparable usefulness of the two. Another subgroup analysis run on the basis of the presumed iodine status of the participants also yielded nonsignificant results.

**Conclusion:**

There is a higher incidence of thyroid cancer among patients of STN, however, given the low quality of existing evidence on the topic, it is crucial to conduct larger studies that can establish association with a greater precision.

**Supplementary Information:**

The online version contains supplementary material available at 10.1186/s13044-022-00140-6.

## Introduction

Thyroid nodules are discrete lesions of the thyroid parenchyma with a comparatively low yet significant potential to develop malignancy. They are a common clinical finding, usually encountered incidentally [1], with prevalence ranging from 2 to 6% for clinically palpable nodules and 19 to 35% for ultrasonographically detected nodules [2]. The incidence is even higher on surgery or autopsy [2] .

The risk of malignancy among thyroid nodules has been estimated to range from 7 to 15% [3], high enough to warrant appropriate diagnostic means where carcinoma suspicion is present. Various clinical practice elements are known to predict the risk of malignancy. Female gender and radiation exposure seem to increase the probability of developing cancerous nodules [4]. Although older literature suggested a bimodal distribution of the risk of progression to carcinoma i.e. both young and old ages being associated with a higher risk of progression to carcinoma [5–7], newer literature suggests a decreasing general malignancy rate of thyroid nodules with advancing age [8, 9]. Recent advances in the understanding of thyroid nodules also point to their location as an independent predictor of malignancy risk, with mid-lobar, upper pole and lateral nodules carrying the highest likelihood of progression to carcinoma [10, 11]. It is also to be noted that cold nodules are at a much greater risk of developing malignancy as compared to hot nodules [12].

The term goiter refers to an abnormal growth or increase in size of the thyroid gland which may result from a single nodule or multiple nodules. Multinodular Goiter (MNG) has historically been considered a benign condition with a low risk of malignancy, however, this idea has been called into question after numerous studies have reported an incidence of carcinoma in MNG approaching that of a Single Thyroid Nodule (STN) [13–16], and at times even exceeding it [17]. Contradictory results from various studies exploring comparative risk of carcinoma in STN and MNG merit the conduction of a meta-analysis to adequately answer this question. As the risk of malignancy dictates diagnostic evaluation and management of thyroid nodules, the assessment of carcinoma risk holds significant clinical value. The only previous meta-analysis addressing this research question was published in 2013 [18], however, that too was limited by a smaller sample size and some statistical errors. It also excluded studies pertaining to important demographics such as children, and thus lacks a comprehensive picture that this meta-analysis promises.

## Material and methods

This systematic review conforms to the guidelines elucidated in *Preferred Reporting Items for Systematic Reviews and Meta-Analyses* (PRISMA) statement [19] and has been registered with The International Prospective Register of Systematic Reviews PROSPERO (CRD42021284103).

### Eligibility criteria

#### Types of studies

The study designs eligible for inclusion in our systematic review were observational studies (cross sectional, cohort, case–control) and Randomized Controlled Trials (RCTs). However, we did not find RCT evidence catering to our topic. No language or location restrictions were applied. Databases were searched from conception to the present and non-English articles were translated to extract the pertinent information.

#### Types of participants

Studies which reported patients having diagnoses of MNG or STN through either fine needle aspiration biopsy (FNAC) or histopathologically via surgical intervention were included.

Studies in which cancer diagnosis was made clinically, ultrasound alone, or those reporting cancer prevalence in patients with toxic (hot) nodules were excluded.

#### Types of comparisons

In our analysis, we compared the risk of thyroid cancer following MNG and STN with each other.

#### Types of outcomes

Our primary outcome was the prevalence of thyroid cancer among patients with MNG and STN.

### Data sources and search strategy

We searched the following sources from inception to August 2021.

• Electronic databases: MEDLINE (via PubMed), Cochrane Database of Systematic Reviews (CDSR), Science Direct.
• International Trial Registers: International Clinical Trials Registry Platform (ICTRP), ClinicalTrials.gov.
• Grey Literature sources: Google Scholar, Grey Literature Report and Virtual Healthy Library.

A combination of keywords and Mesh terms like “Multinodular goiter”, “Goiter, Nodular’’, “Thyroid Neoplasms” was used to search the databases. The complete search strategy of MEDLINE is provided in the supplementary file. The same search strategy was followed for the other databases. No filter of language, time and study design was used in order to retrieve the maximum literature. We also manually sieved the reference lists of retrieved articles and previous reviews to identify any missed studies, and contacted authors of the respective articles for any missing information vital to our review (Additional file 1).

#### Study selection and data extraction

All the literature search results were uploaded to Mendeley, and after de-duplication of articles, two reviewers independently performed screening on the basis of title and abstracts. Full text screening was done for the remaining articles and only those studies that met the predefined eligibility criteria were included. Two reviewers independently extracted the following data items from each study: type of study design, country where the study was performed, sample size of the study, total number of patients, age, sex, type of nodular goiter (MNG vs STN), length of follow up, prevalence of thyroid cancer, diagnosis of cancer (through surgery or FNAC), type of thyroid cancer, indication for surgery, family history of thyroid cancer, history of radiation exposure, and histopathology results. Any disagreement between the two reviewers was resolved through mutual discussion. A PRISMA flowchart is constructed to illustrate the study selection process.

#### Risk of bias in individual studies

Methodological quality of our included studies was assessed by two authors independently using Newcastle Ottawa Scale (NOS) for cohort studies [20]. Studies were allocated stars on the basis of three perspectives: the selection of the study groups; the comparability of the groups; and the ascertainment of outcome of interest. A modified NOS scale was used for evaluating the quality of shortlisted cross-sectional studies. A third reviewer resolved any conflict that arose between the two reviewers regarding quality assessment.

#### Assessment of heterogeneity

We assessed heterogeneity among the studies included in our analysis using the Chi-square test. Values were interpreted according to the *Cochrane Handbook for Systematic Reviews of Interventions*, Sect. 10.10 [21]. Significance level was set at p value less than 0.10. Inconsistency was quantified using the I^2^ index. I^2^ > 50 percent constitutes a major inconsistency.

#### Assessment of reporting biases

Our meta-analysis consisted of more than 10 studies, so we constructed a funnel plot and subjected it to visual inspection to assess the presence of reporting bias. However, asymmetry can also be due to some other causes like true heterogeneity or presence of publication bias.

#### Statistical analysis

We performed meta-analyses using Review Manager (RevMan) (version 5.4. Copenhagen: The Nordic Cochrane Center, The Cochrane Collaboration, 2014). We used the DerSimonian and Laird random-effect model for conducting our meta-analysis. Prevalence of thyroid cancer in patients with MNG was compared to the prevalence of thyroid cancer in patients with STN. Pooled odds ratio with 95 percent confidence interval was calculated.

#### Additional analyses

We aimed to perform subgroup analyses based on the type of diagnostic method (surgery vs FNAC), history of thyroid cancer in family, history of radiation exposure and iodine status of the locale where the study was conducted. WHO data on iodine status of different locales worldwide was used to run this subgroup analysis [22] .

#### Confidence in cumulative evidence

The certainty of evidence was assessed using the GRADE (*Grading of Recommendations Assessment, Development, and Evaluation*) assessment tool [23]. The GRADE approach characterizes the quality of evidence in one of the four grades: high (true effect lies close to that of the estimate of the effect), moderate (true effect is likely to be close to the estimate of the effect, but there is a possibility that it is substantially different), low (true effect may be substantially different from the estimate of the effect), and very low (true effect is likely to be substantially different from the estimate of effect). Quality of evidence of our pooled estimate was rated down for limitations in study design or execution (risk of bias), inconsistency of results, indirectness of evidence, imprecision, and publication bias (Additional file 2).

## Results

### Study selection

**Fig. 1:**
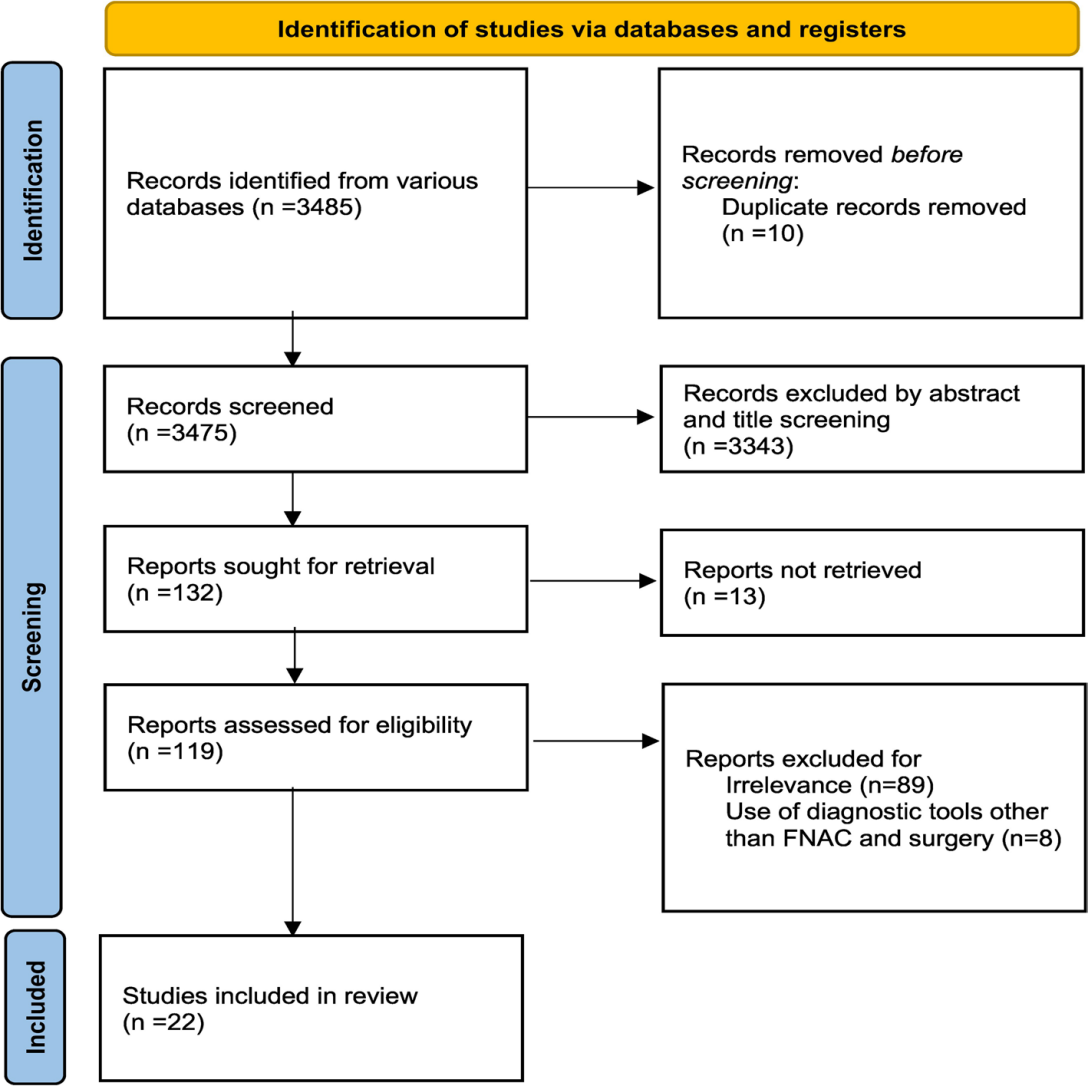
PRISMA Flow Diagram

We identified 3485 records through a comprehensive database search. After removal of duplicates (*n* = 10), 3475 records were screened on the basis of titles and abstracts. 3343 records were excluded through screening of title and abstract according to the eligibility criteria. 13 records were excluded as their full texts could not be retrieved. The remaining 119 articles were assessed for full text eligibility. 89 studies were found to be irrelevant, and were thus excluded. 8 articles were excluded because they used diagnostic criteria other than FNAC and surgery. The remaining 22 studies were included in this systematic review (Fig. 1).

### Study and patient characteristics

We included 22 studies spanning from 1992 to 2018 in our meta-analysis after extensive literature review [3, 12, 15, 16, 24–41]. 50,321 patients were included in the study. 22,352/50321 (44.42%) patients were enrolled in the STN sub-group and 27,864/50321(55.37%) patients in the MNG group. Most common cancer type was found to be papillary thyroid cancer, followed by follicular and medullary thyroid cancer. Nearly half of the studies subjected to meta-analysis used surgical intervention for diagnostic purposes and the rest used FNAC. Thyroidectomy was performed in the majority of the studies. Highest number of included studies from a single country (6/22) were from Italian demography followed by Turkey, Saudi Arabia and USA (Tables 1 and 2).

**Table 1:** Characteristics of Included Studies

Author	Year of publication	Study design	Region	Iodine deficiency	Mean age	Gender distribution
Abu Eshy, SA et al	1995	Retrospective Cohort	Saudi Arabia	No	STN, 34.7 ± 14.2 y MNG, 37.7 ± 14.3 y	84.5% F, 15.5% M
Ajarma, Khalid Y et al	2018	Cross sectional	Jordan	No	44.3 ± 14.5 y	77.8% F, 22.2% M
Belfiore, Antonino et al	1992	Cross sectional	Italy	Yes	-	89% F, 11% M
Deandrea et al	2001	Retrospective Cohort	Italy	Yes	51 y	96.7% F, 3.3% M
Dirikoc, A et al	2017	Cross sectional	Turkey	Yes	49.09 ± 11.93 y	80% F, 20% M
Edino ST et al	2010	Cross sectional	Nigeria	No	38.8 y	-
Franklyn JA et al	1993	Prospective cohort	UK	-	-	-
Frates et al	2006	Retrospective cohort	USA	No	46.2 ± 0.9 y	87.8% F, 12.2% M
Kaliszewski, Krzysztof et al	2016	Retrospective cohort	Poland	Yes	53.0 ± 16.4 y	-
Khairy et al	2004	Cross sectional	Saudi Arabia	No	-	-
Marqusee et al	2000	Cross sectional	USA	No	46.3 y	91% F, 9% M
Matesa et al	2005	Retrospective cohort	Croatia	No	-	93% F, 7% M
Miccoli, Paolo et al	2006	Prospective cohort	Italy	Yes	49.5 y	70% F, 30% M
Nobrega et al	2007	Retrospective cohort	Brazil	No	47.9 ± 15.2 y	92% F, 8% M
Papendieck et al	2015	Prospective Cohort	Argentina	-	< 19 y	-
Papini et al	2002	Cross sectional	Italy	Yes	47.8 ± 13.3 y	87% F, 13% M
Provenzale et al	2016	Retrospective cohort	Italy	Yes	50.1 ± 13.8 y	70% F, 30% M
Rago et al	2010	Cross sectional	Italy	Yes	M, 50 ± 17 y; F, 48 ± 23 y	81% F, 19% M
Rios et al	2018	Prospective cohort	Spain	No	51.9 ± 16.7 y	86% F, 14% M
Sachmechi et al	2000	Retrospective cohort	USA	No	52 y	92.5% F, 7.5% M
Sippel et al	2007	Retrospective cohort	USA	No	47 y	81.5% F, 18.5% M
Taneri et al	2005	Cross sectional	Turkey	Yes	47.2 y	81% F, 19% M

*Abbreviations: STN *Solitary thyroid nodule*, MNG *Multinodular goitre*, F *Female*, M *Male*, y *Years*, (-) *Data not reported

**Table 2:** Diagnostic Findings of Included Studies

Author	Total patients	Diagnostic method	Diagnosis		Thyroid carcinoma	Varieties of thyroid carcinoma		
						**Papillary (%)**	**Follicular (%)**	**Anaplastic (%)**
Abu Eshy, SA et al	277	Surgery	STN	105 (38%)	16/105 (15.2%)	78.6	14.3	7.1
			MNG	172 (62%)	14/172 (8%)	86	0	0
Ajarma, Khalid Y et al	567	Surgery	STN	224 (39.5%)	92/224 (41.1%)	62	17.4	3.3
			MNG	343 (60.5%)	100/343 (29.2%)	55	15	2
Belfiore, Antonino et al	5637	FNAC	STN	4485 (79.5%)	212/4485 (4.7%)	68	23	9
			MNG	1152 (20.5%)	47/1152 (4.1%)	-	-	-
Deandrea et al	420	FNAC	STN	246 (58.5%)	15/246 (6.1%)	-	-	-
			MNG	174 (41.5%)	12/174 (6.9%)	-	-	-
Dirikoc, A et al	1719	Surgery	STN	227(13.2%)	105/227 (46.3%)	84.2	3.8	1.5
			MNG	1492(86.8%)	618/1492 (41.4%)	94.4	1.9	0.3
Edino ST et al	173	Surgery, FNAC	STN	13(7.5%)	1/13(7.6%)	40	52	4
			MNG	160(92.5%)	24/160 (15%)	-	-	-
Franklyn JA et al	393	FNAC	STN	321 (81.7%)	19/321(5.9%)	-	-	-
			MNG	72 (18.3%)	1/72 (1.4%)	-	-	-
Frates et al	1985	FNAC	STN	1181 (59.5%)	175/1181 (14.8%)	86.3	12	-
			MNG	804 (40.5%)	120/804 (14.9%)	91.7	5	-
Kaliszewski, Krzysztof et al	1645	Surgery	STN	493 (30%)	98/493 (19.9%)	78.5	8.2	6.1
			MNG	1152 (70%)	68/1152 (5.9%)	89.7	5.8	2.9
Khairy et al	296	Surgery	STN	172 (58%)	24/172 (14%)	91.6	4.2	4.2
			MNG	124 (42%)	16/124 (12.9%)	77	23	-
Marqusee et al	150	FNAC	STN	60 (40%)	4/60 (6.7%)	-	-	-
			MNG	90 (60%)	8/90 (8.9%)	-	-	-
Matesa et al	406	FNAC	STN	117(29%)	6/117 (5.1%)	100	-	-
			MNG	289(71%)	15/289 (5.2%)	53.3	-	6.67
Miccoli, Paolo et al	510	Surgery	STN	69 (13.5%)	1/69(1.4%)	-	-	-
			MNG	441 (86.5%)	63/441 (14.3%)	-	-	-
Nobrega et al	220	Surgery	STN	72 (32.7%)	27/72 (37.5%)	9.5	-	-
			MNG	148 (67.3%)	47/148 (31.8%)	-	-	-
Papendieck et al	62	Surgery	STN	45 (72.6%)	7/45 (15.6%)	100	-	-
			MNG	17 (27.4%)	7/17 (41.2%)	100	-	-
Papini et al	402	US guided FNAC	STN	195 (48.5%)	18/195 (9.2%)	87	6.5	-
			MNG	207(51.5%)	13/207 (6.3%)	-	-	-
Provenzale et al	665	Surgery	STN	259 (39%)	59/259 (22.8%)	100	-	-
			MNG	406 (61%)	100/406 (24.6%)	100	-	-
Rago et al	33,472	FNAC	STN	13,549 (40.5%)	531/13549 (3.9%)	84	-	-
			MNG	19,923 (59.5%)	471/19923 (2.4%)	87.3	-	-
Rios et al	221	Surgery	STN	169 (76.5%)	29/169 (17.12%)	81.25	3.125	3.125
			MNG	52 (23.5%)	3/52 (5.7%)	-	-	-
Sachmechi et al	142	FNAC	STN	50 (35.2%)	4/50 (8%)	50	50	-
			MNG	92 (64.8%)	9/92 (9.78%)	33.33	33.33	-
Sippel et al	295	FNAC	STN	132 (45%)	37/132 (28%)	19.3	57	6.3
			MNG	163 (55%)	27/163 (16.6%)	-	-	-
Taneri et al	370	Surgery	STN	133 (25.6%)	24/133 (18.04%)	-	-	-
			MNG	237 (45.7%)	35/237 (14.64%)	-	-	-

*Abbreviations: FNAC *Fine needle aspiration cytology*, STN *Solitary thyroid nodule*, MNG *Multinodular goiter*, (-) *Data not reported

### Quality assessment

Newcastle Ottowa Scale (NOS) was used to assess risk of bias in 13 cohort studies. 4/13 (30.8%) were prospective cohorts and 9/13 (69.2%) studies were retrospective cohorts. Out of 13 included cohort studies, 2/13(15.4%) had a low risk of bias and 11/13 (84.6%) had moderate risk. 11/13(84.6%) cohort studies didn’t assess comparability which contributed to an increased risk of bias. We used modified NOS to find out the risk of bias in 9 cross-sectional studies included in our meta-analysis. 3/10 (30%) cross-sectional studies reported low risk of bias and 7/10 (30%) had moderate risk. Only 3 cross-sectional studies performed comparability analysis. All included studies had representative samples, ascertainment of exposure and negligible non-respondent numbers (Additional file 3).

### Risk of thyroid cancer in patients with Multinodular Goiter (MNG) vs Solitary Thyroid Nodule (STN)

**Fig. 2:**
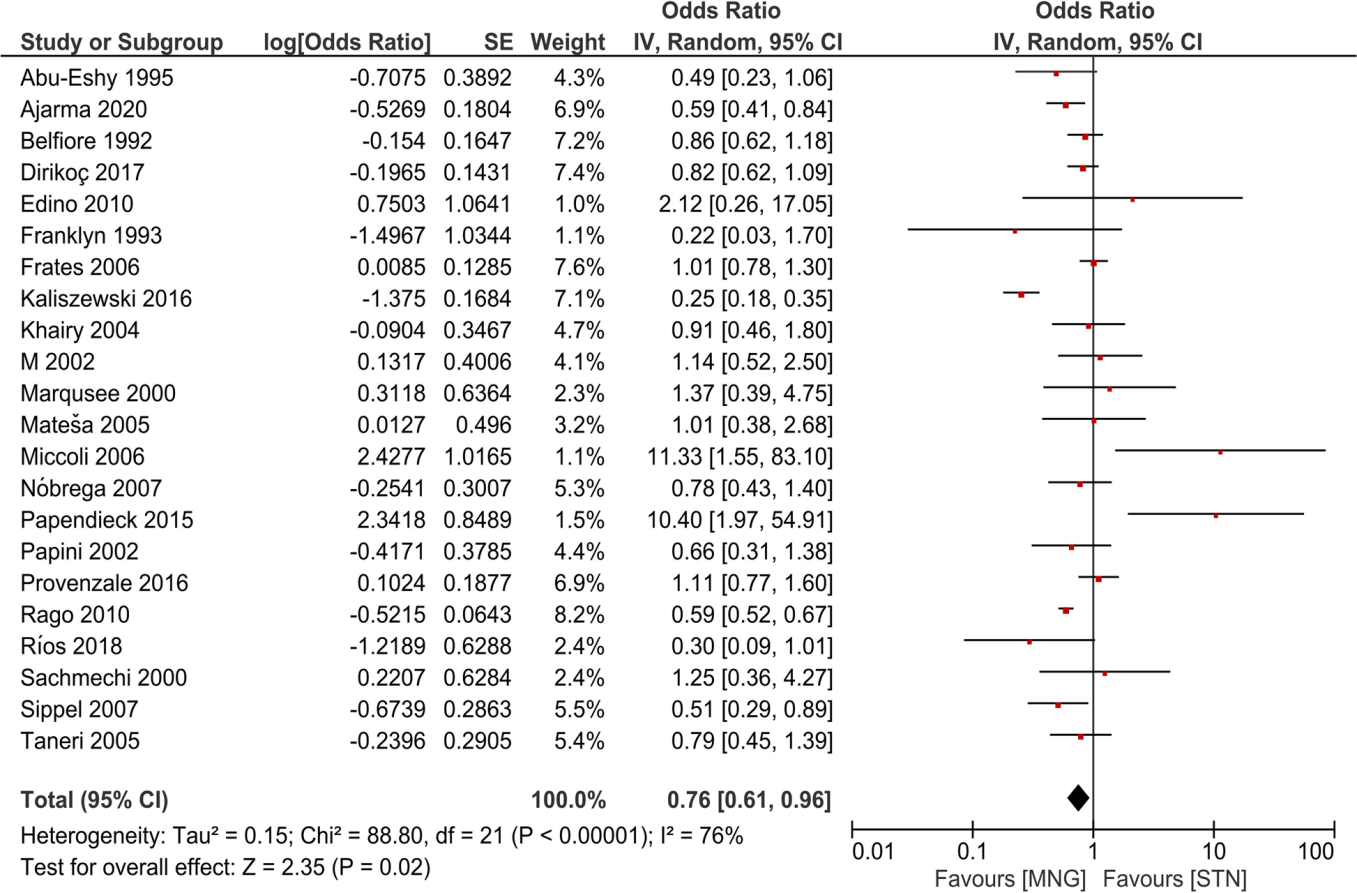
Forest plot for risk of thyroid cancer in patients with Multinodular Goiter (MNG) vs. Solitary Thyroid Nodule (STN)

We constructed a forest plot using the random effect model for meta-analysis. The risk of thyroid cancer was found to be significantly lower in MNG as compared to STN. Summary OR value was calculated to be 0.76 (CI:0.61–0.96), with significant inconsistency across studies [I^2^ = 76%] (Fig. 2).

### Subgroup analyses

**Fig. 3:**
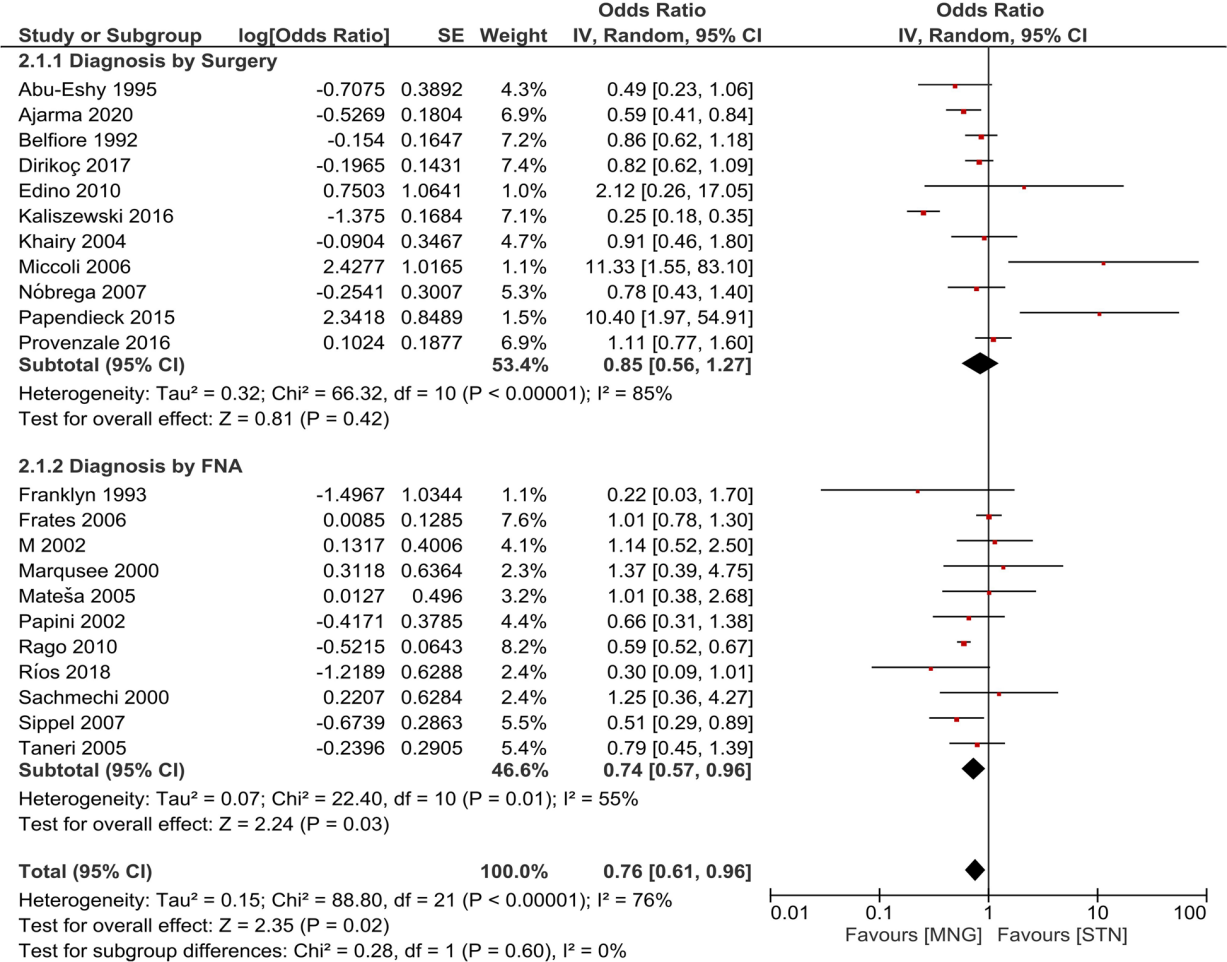
Subgroup analysis on the basis of diagnostic methods

We performed subgroup analyses on the basis of diagnostic method and the iodine status in the locale to explore the causes of heterogeneity. 11/22 (50%) of our included studies used surgical resection as the diagnostic method while the other 11/22 (50%) diagnosed thyroid cancer via FNAC. On running the subgroup analysis, no significant differences between the two groups were observed (Fig. 3).

**Fig. 4:**
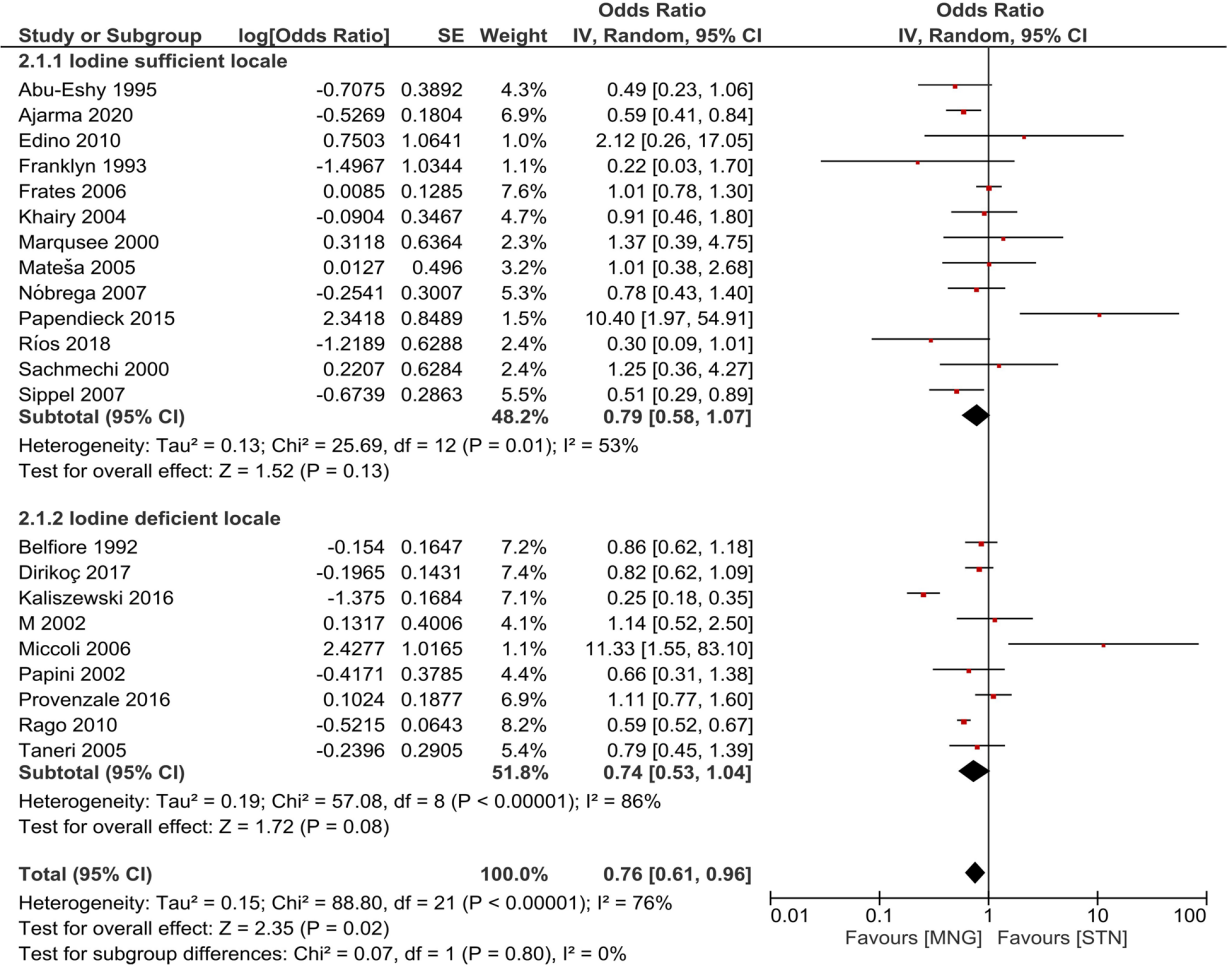
Subgroup analysis on the basis of iodine status in the locale

Similarly, subgroup analysis on the basis of iodine status in the locale reported an insignificant difference between the two groups. Insufficient data was available to perform subgroup analysis on the basis of history of thyroid cancer in the family and history of radiation exposure (Fig. 4).

### Assessment of reporting bias

**Fig. 5:**
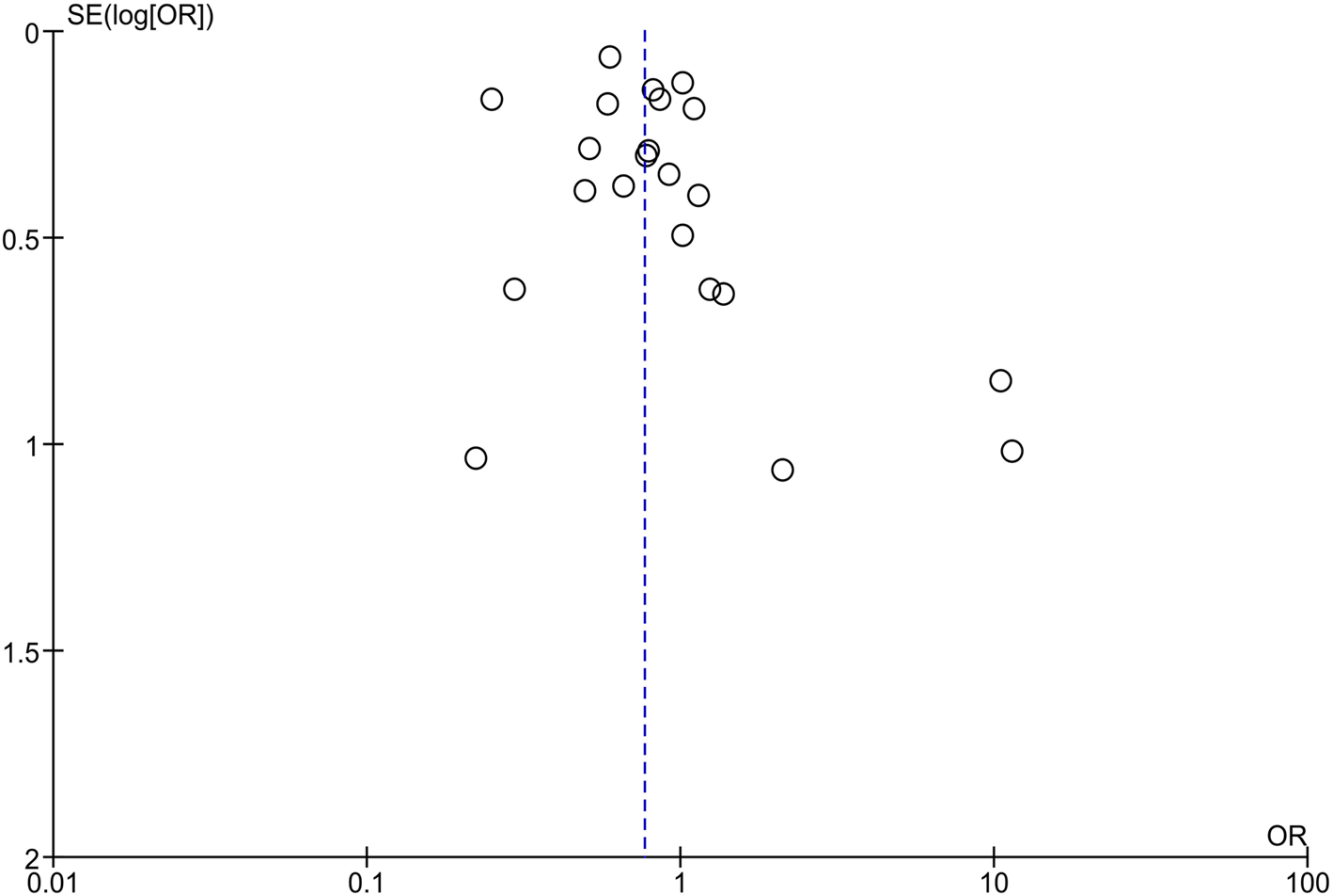
Funnel plot for assessment of reporting bias

A funnel plot for these 22 studies was subjected to visual inspection. Minor asymmetry was observed which confirmed insignificant publication bias (Fig. 5).

## Discussion

Both solitary thyroid nodules (STN) and multinodular goiter (MNG) usually present with a single nodule on palpation because the dominant nodule in MNG obscures the detection of other smaller nodules [42, 43]. A more substantial problem, however, arises when the results of cytological evaluation are indeterminate, and physicians are left withsurgery as the only option to definitively diagnose any malignancy. However, given that surgical evaluation for all cases of indeterminate thyroid nodules is neither clinically possible nor recommended, it is imperative to establish variables such as nodularity as risk factors for malignancy in order to better clinically assess individual patient risk for cancer [44]. It has been estimated that if surgery is performed for all indeterminate cases of FNAC, thyroid cancer will be found in only 10–40% of the cases[45], making the rest of the surgeries needless and futile. It is therefore essential to preemptively predict the risk of carcinoma in patients based on their clinical characteristics and examination findings, particularly nodularity. This will help formulate standard guidelines that can aid clinical decision making and management.

Our analysis corroborated the previously held view that single thyroid nodules are associated with a higher risk of thyroid carcinoma than multinodular goiter [18], and hence can be considered an independent risk factor to be used for carcinoma risk stratification. The purpose of thyroid nodule evaluation, therefore, is to identify both, nodules that may potentially be malignant and toxic nodules which are known to carry a lower risk of malignancy [46]. Such risk stratification allows to avoid histological evaluation, which is both needless and invasive, in cases of indeterminate thyroid nodules.

However, given the emerging evidence of equal or even greater carcinoma risk in MNG in some of the more recent studies [12, 16, 47], our findings can be attributed to several factors or limitations. Firstly, it has been estimated that 23% of clinically diagnosed solitary nodules are in fact dominant nodules within MNG [48], which if accounted for, would substantially increase not only the incidence of MNG, but also the attributed risk of thyroid carcinoma in MNG. The detection of thyroid carcinoma has increased with the development of better diagnostic tools [49], therefore, the incidence of carcinoma in MNG is expected to rise proportionally.

Moreover, conventionally only the dominant nodule has been biopsied in an MNG until the recent updates in guidelines, imposing a limitation on this study and any such meta-analysis carried out in the future. Frates et al. found that biopsying only the largest nodule carries with it the risk of missing a thyroid carcinoma by 15% [15]. In effect, although the dominant nodule in MNG carries a comparatively greater risk of progression to thyroid carcinoma [50], the rest of the nodules carry enough risk to be separately treated as solitary nodules on their own. Evidence also increasingly suggests that although cancer risk per nodule decreases with multinodular goiter, the cumulative risk when adjusted for the number of nodules equals that of a solitary nodule [51].

Unquestionably, the most important finding in this regard however, comes from Kaliszewski et al. [28]. FNAC was found to be three times more likely to give false negatives in the setting of MNG compared to STN, owing primarily to biopsy of only a specific nodule in MNG or collection of nondiagnostic samples. This is part of the reason why MNG is associated with higher reoperation rates than STN [28]. Given that FNAC is the tool most commonly employed for diagnosis of malignancy, this becomes an important confounding factor contributing to a lower than expected incidence of cancer in MNG.

A subgroup analysis was conducted on the basis of diagnostic methods in order to compare histological diagnosis (surgery) with cytological diagnosis (FNAC). Although surgery has long been considered the gold standard for diagnosis of malignancy in the thyroid, our results found no statistically significant difference in the effectiveness of the two. However, a significantly higher incidence of nodules on autopsy [52] poses a conundrum as visualization of a higher number of nodules would automatically expand the likelihood of a carcinoma diagnosis in surgery. Therefore, the insignificance of results were likely due to FNAC being carried out on only those nodules that have already been found to have malignant characteristics by ultrasound, a phenomenon thus termed ‘FNAC enrichment’.

Moreover, another subgroup analysis based on the iodine intake status of the participants also yielded statistically nonsignificant results, indicating that iodine intake had a minimal effect on progression to carcinoma. This differs from the previous meta-analysis which suggests that a difference in cancer risk between MNG and STN in different populations may stem from iodine intake difference at different locations [18].

Although further studies, particularly prospective, are crucial in establishing nodularity as a reliable predictor of malignancy, it is imperative to note the implications of this meta-analysis on existing understanding of thyroid nodules, especially the Thyroid Imaging, Reporting and Data System (TI-RADS), a parallel system of malignancy risk stratification that relies entirely on ultrasonographic features [53] . Patients with a lower malignancy risk determined clinically by physicians based on existing evidence on nodularity, may only be required to undergo ultrasonography, thus minimizing the need for invasive procedures such as FNAC or surgery, while also improving accuracy and sensitivity. This may even render nodularity only of an auxiliary importance in the prediction of malignancy risk.

### Strengths and limitations

The strengths of this study lie in the extensive literature search, the inclusion of all demographics including children that had been excluded in previous literature, rigorous quality assessment and minimal reporting bias. Given the scarcity of literature on the topic, this meta-analysis not only presents the largest pooling of data on the subject till date but also points out the gaps in existing literature.

Limitations of this meta-analysis arise primarily from the limited prospective data to dictate therapy. Most of the studies on the topic are observational studies, and hence are ill equipped to establish correlation. Moreover, most of the included studies are retrospective which is a major source of potential selection bias i.e. solitary nodules are more likely to be submitted for FNAC than MNG. This may further contaminate the results in favor of cancer risk in solitary nodules. Secondly, important demographic data such as age and gender as well as a distinction between incidental and non-incidental discovery of microcarcinomas were inconsistently reported, hindering any attempt to run a subgroup analysis on their basis and establishing a trend. Lastly, some of the studies did not describe any inclusion or exclusion criteria and may have followed selection criteria slightly differing from this review, thus polluting the overall sample.

## Conclusions

Solitary thyroid nodules were found to carry a greater risk of thyroid carcinoma compared to multinodular goiter, however, the validity and strength of this association are questionable owing to the low quality of existing literature on the topic.

## Supplementary Information

**Additional file 1.** Search Strategy.

**Additional file 2:** **GRADE.** We used the GRADE assessment tool to assess the quality of evidence. Studies being observational in nature, inconsistency and serious risk of bias contributed to decreased quality.

**Additional file 3.** Critical Appraisal of Cohort studies.

## Data Availability

All data generated or analyzed during this study are included in this published article (and its supplementary information files).
