# Correction to: Solitary and multiple thyroid nodules as predictors of malignancy: a systematic review and meta‑analysis

**DOI:** 10.1186/s13044-023-00159-3

**Published:** 2023-05-08

**Authors:** Aqeeb Ur Rehman, Muhammad Ehsan, Haseeba Javed, Muhammad Zain Ameer, Aleenah Mohsin, Muhammad Aemaz Ur Rehman, Ahmad Nawaz, Zunaira Amjad, Fatima Ameer

**Affiliations:** 1 Department of Medicine, King Edward Medical University, Lahore, Pakistan; 2 Clinical Research Fellow, Massachusetts General Hospital, Harvard Medical School, Boston, USA; 3 Department of Medicine, Services Institute of Medical Sciences, Lahore, Pakistan; 4 Department of Medicine, Mayo Hospital, Lahore, Pakistan

Correction to: *Thyroid Res***15**, 22 (2022)

10.1186/s13044-022-00140-6

Following publication of the original article [1], the authors identified an error in the affiliation of Fatima Ameer.

The incorrect affiliations are: Fatima Ameer^2,4^

The correct affiliation is: Fatima Ameer^4^

^4^Department of Medicine, Mayo Hospital, Lahore, Pakistan

The affiliation has been updated above and the original article [1] has been corrected.
