# Supplementary material for: Solitary and multiple thyroid nodules as predictors of malignancy: a systematic review and meta-analysis
Source: Thyroid Res. 2022 Dec 5;15:22. doi: 10.1186/s13044-022-00140-6 (PMC9720983; doi:10.1186/s13044-022-00140-6)
Supplement: Supplementary file 1 — Additional file 1. Search Strategy. [file 13044_2022_140_MOESM1_ESM.docx]

**Search Strategy:**

| **Number** | **Search Terms** |
| --- | --- |
| #1 | Multinodular goiter [All Fields] |
| #2 | Multinodular goitre [All Fields] |
| #3 | MNG [All Fields] |
| #4 | "Goiter, Nodular"[Mesh] |
| #5 | #1 OR #2 OR #3 OR #4 |
| #6 | Solitary thyroid nodule [All Fields] |
| #7 | Single nodul* [All Fields] |
| #8 | STN [All Fields] |
| #9 | #6 OR #7 OR #8 |
| #10 | Thyroid cancer [All Fields] |
| #11 | Thyroid carcinoma [All Fields] |
| #12 | "Thyroid Neoplasms"[Mesh] |
| #13 | "Thyroid Cancer, Papillary"[Mesh] |
| #14 | #10 OR #11 OR #12 OR #13 |
| #15 | #5 AND #9 AND #14 |
