# Supplementary material for: Solitary and multiple thyroid nodules as predictors of malignancy: a systematic review and meta-analysis
Source: Thyroid Res. 2022 Dec 5;15:22. doi: 10.1186/s13044-022-00140-6 (PMC9720983; doi:10.1186/s13044-022-00140-6)
Supplement: Supplementary file 2 — Additional file 2: GRADE. We used the GRADE assessment tool to assess the quality of evidence. Studies being observational in nature, inconsistency and serious risk of bias contributed to decreased quality. [file 13044_2022_140_MOESM2_ESM.docx]

**GRADE:**

We used the GRADE assessment tool to assess the quality of evidence. Studies being observational in nature, inconsistency and serious risk of bias contributed to decreased quality.

| **Outcome** | **Study (n)** | **Risk of bias** | **Inconsistency** | **Indirectness** | **Imprecision** | **Publication Bias** | **Strength of evidence** |
| --- | --- | --- | --- | --- | --- | --- | --- |
| Risk of Thyroid cancer | 22 | Serious | Serious | Not serious | Not serious | Not serious | ⊕⊝⊝⊝  Very Low |
