# Supplementary material for: Solitary and multiple thyroid nodules as predictors of malignancy: a systematic review and meta-analysis
Source: Thyroid Res. 2022 Dec 5;15:22. doi: 10.1186/s13044-022-00140-6 (PMC9720983; doi:10.1186/s13044-022-00140-6)
Supplement: Supplementary file 3 — Additional file 3. Critical Appraisal of Cohort studies. [file 13044_2022_140_MOESM3_ESM.docx]

| **Critical Appraisal of Cohort studies** | | | | | | | | | | | |
| --- | --- | --- | --- | --- | --- | --- | --- | --- | --- | --- | --- |
| **Study** | **Type** | **Representativeness (1)** | **Selection of Non exposed Cohort (1)** | **Exposure (1)** | **Outcome of Interest (1)** | **Comparability (2)** | **Assessment of Outcome (1)** | **Follow-up Long Enough (1)** | **Adequacy of Follow-ups (1)** | **TOTAL (9)** | **Risk of bias** |
| Deandrea | Cohort (retrospective) | 1 | 1 | 1 | 1 | 0 | 1 | 1 | 1 | 7 | Moderate |
| Franklyn JA | Prospective Cohort | 1 | 1 | 1 | 1 | 0 | 1 | 1 | 1 | 7 | Moderate |
| Frates | Retrospective Cohort | 1 | 1 | 1 | 1 | 1 | 1 | 1 | 0 | 8 | Low |
| Kaliszewski, Krzysztof | Retrospective cohort | 1 | 1 | 1 | 1 | 0 | 1 | 1 | 1 | 7 | Moderate |
| Matesa | Retrospective Cohort | 1 | 1 | 1 | 1 | 0 | 1 | 0 | 0 | 5 | Moderate |
| Nobrega | Retrospective Cohort | 1 | 1 | 1 | 1 | 0 | 1 | 1 | 1 | 7 | Moderate |
| Papendieck | Prospective Cohort | 1 | 1 | 1 | 1 | 2 | 1 | 1 | 1 | 9 | Low |
| Provenzale | Retrospective Cohort | 1 | 1 | 1 | 1 | 0 | 1 | 1 | 1 | 7 | Moderate |
| Sippel | Retrospective Cohort | 1 | 1 | 1 | 1 | 0 | 1 | 1 | 1 | 7 | Moderate |
| Sachmechi | Retrospective Cohort | 1 | 1 | 1 | 1 | 0 | 1 | 1 | 0 | 6 | Moderate |
| Abu Eshy | Retrospective Cohort | 1 | 1 | 1 | 1 | 0 | 1 | 1 | 1 | 7 | Moderate |
| Rios | Prospective Cohort | 1 | 1 | 1 | 1 | 0 | 1 | 1 | 1 | 7 | Moderate |
| Miccoli, Paolo | Prospective Cohort | 1 | 1 | 1 | 1 | 0 | 1 | 1 | 0 | 6 | Moderate |

| **Critical Appraisal of Cross sectional studies** | | | | | | | | | | |
| --- | --- | --- | --- | --- | --- | --- | --- | --- | --- | --- |
| **Study** | **Type** | **Representativeness of the sample (1)** | **Sample size (1)** | **Non-respondents (1)** | **Ascertainment of the exposure (2)** | **Comparability (2)** | **Assessment of the outcome (2)** | **Statistical test (1)** | **TOTAL (10)** | **Risk of bias** |
| Ajarma, Khalid Y | Cross Sectional | 1 | 1 | 1 | 2 | 2 | 2 | 1 | 10 | Low |
| Dirikoc, A | Cross Sectional | 1 | 1 | 1 | 2 | 0 | 2 | 1 | 8 | Moderate |
| Edino ST | Cross Sectional | 1 | 1 | 1 | 2 | 0 | 2 | 1 | 8 | Moderate |
| Marqusee | Cross Sectional | 1 | 1 | 1 | 2 | 0 | 2 | 0 | 7 | Moderate |
| Rago | Cross Sectional | 1 | 1 | 1 | 2 | 2 | 2 | 1 | 10 | Low |
| Khairy | Cross Sectional | 1 | 1 | 1 | 2 | 0 | 2 | 1 | 8 | Moderate |
| Papini | Cross Sectional | 1 | 1 | 1 | 2 | 2 | 2 | 1 | 10 | Low |
| Taneri | Cross Sectional | 1 | 1 | 1 | 2 | 0 | 2 | 0 | 7 | Moderate |
| Belfiore, Antonino | Cross Sectional | 1 | 1 | 1 | 2 | 0 | 2 | 1 | 8 | Moderate |
